# Are parental dietary patterns associated with children’s overweight and obesity in China?

**DOI:** 10.1186/s12887-020-1910-z

**Published:** 2020-01-11

**Authors:** Daisheng Tang, Tao Bu, Xuefan Dong

**Affiliations:** School of Economics and Management, Beijing Jiaotong University, Beijing, 100044 China

**Keywords:** Parental food intake, Dietary patterns, children’s overweight and obesity, children’s age, Areas distribution

## Abstract

**Background:**

It is believed that parents have a great influence on their children’s dietary behaviours. However, it is not clear whether parental food patterns are associated with children’s nutritional status in China, which includes a vast territory with rich, diverse cultures. The goal of this project is to systematically study the associations between parental food intake and children’s overweight and obesity in China, according to children’s ages and regional differences.

**Methods:**

Based on individual food consumption data from the China Health and Nutrition Survey (CHNS) package in 2011, cross-sectional studies have previously been conducted to analyse the association between different categories of food intake of parents and children. The current study extends this research by directly.

**Results:**

Our analysis results show that parental food intake is highly correlated with children’s food intake, with the estimated coefficients of most food intake categories being greater than 0.5. Furthermore, this association between parental food intake and children’s overweight and obesity is most significant in young children, but it begins to weaken in relation to children aged between 13 and 18. Additionally, the associations between parental food intake and children’s overnutrition are more significant in rural areas than they are in urban areas.

**Conclusions:**

The association between parental food intake and childhood overweight and obesity is significant, although it varies considerably according to food categories, children’s ages and area differences. These results show promise for intervening in the overnutrition of children by controlling household dietary patterns according to children’s developmental stages and regional differences.

## Background

In the past few decades, the number of children who are overweight or obese has grown explosively. From 2002 to 2015, the percentage of Chinese children who were overweight increased from 4.5 to 9.6%, and the percentage of those who were obese increased from 2.1 to 6.4% [1]. Many empirical studies have confirmed that obesity in adolescence increases the risk of various diseases in middle age [2]. Therefore, it is vital to study the factors influencing Chinese children’s nutritional status in order to improve it.

It is widely known that parents have a significant influence on their children’s dietary behaviours and nutritional status [3]. Bandura’s [4] cognitive theory emphasises that the role of the perceived social environment is crucial for maintaining healthy eating behaviours; as such, social environmental variables (e.g., home, schools and neighbourhoods) play an important role in children’s healthy development [5–7]. Evidence suggests that parental eating behaviours are particularly important for the development of children’s eating behaviours; some studies have shown a high correlation between the dietary patterns of parents and their children [8–10]. Parental role modelling—which includes parental behaviours, attitudes and preferences toward food—has been identified as a potential way in which to influence children’s eating behaviours and nutritional status [11]. This modelling involves an observational learning process that relies on parents to facilitate and encourage healthy behaviour in children, as parents use feeding strategies by deliberately exhibiting preferred eating habits in front of their children to foster healthy eating behaviours. Parental role modelling has been demonstrated to significantly affect children’s healthy eating behaviours, according to both children’s perceptions of it and parental self-reporting [7, 12, 13]. Further, children’s risk of overnutrition can be predicted from a series of eating behaviours self-reported by parents [14].

Another potential way in which parents can cultivate positive eating behaviours in their children is through verbal modelling, in which parents directly emphasise what they consider to be healthy eating behaviours to their children [12]. Parental verbal pressure may include sending positive or negative verbal signals to children about their body mass index (BMI), restricting some types of food and encouraging children to eat less junk food and more fruits and vegetables. Research has demonstrated the positive associations between verbal modelling and children’s eating behaviours [15–17]. However, although parents may think certain eating patterns are beneficial to children, these are not always demonstrated when they select food in real life. Parents may act incongruously, and their environment may influence their food choices, with the consequence that their children’s nutritional status may also be altered in relation to the expected results [7, 18].

If food is freely available, both parental role modelling and verbal pressure modelling have been shown to greatly influence on children’s healthy eating behaviours; however, it has not yet been determined if this influence still exists within the actual Chinese socio-economic structure. China covers a vast territory with an unequal economic structure, and the prevalence of obesity in urban regions is higher than that in rural regions [19, 20]. Urban residents have access to diverse foods and a higher average educational level. In contrast, rural residents’ dietary pattern is relatively unvaried because of the unavailability of diverse foods and the poor infrastructure of rural regions [21]. Further, as children increase in age, they acquire greater autonomy and are subjected to stronger influences from their social environment, including Chinese traditional culture, when they choose their food [22]. Additionally, younger children have a natural preference for eating desserts and snack foods [23]. This may lead parents’ influence on their children’s nutritional status to change as their children grow up. In practice, while parents are gatekeepers for their children’s healthy eating behaviours, children’s eating behaviours are likely influenced by a series of complex factors.

Regional variation and children’s age are the key factors influencing the association of parental dietary patterns with children’s overweight and obesity. However, there have been few comprehensive analyses of the association of parental food patterns and the overweight and obesity of children in different age groups and regions—as well as the trends of this correlation—in China. Previous studies have examined the causal relationship between parental dietary patterns and children’s nutritional status and have shown that a series of parental dietary behaviours have an impact on children’s nutritional status, but it is not clear that parental foods patterns have a significant association with children’s overweight and obesity [7, 9, 10, 14, 24, 25].

Using more detailed distinctions in terms of parents’ food consumption patterns and the food categories they consume in daily life, we directly studied the relationship between parents’ food patterns and children’s overnutrition, taking into consideration the characteristics of Chinese dietary culture. Determining the overweight and obesity rates within children’s different developmental stages and within different regions is very useful in tracking the progression of the overweight and obesity epidemic and in inferring the times and places at which to target intervention strategies for greatest effect. Therefore, the present paper systematically studies the association of parental food patterns with the overweight and obesity of children the ages of 1 and 18 in different areas of China using records of actual parental food consumption. Another related study implemented in the present paper is determining how these associations change according to variations in children’s age and regional differences. This study offers new insights into the establishment of an effective program to address obesity concerns in children.

## Methods

### Data of the China health and nutrition survey (CHNS)

The data used in this study comes from the China Health and Nutrition Survey (CHNS), an ongoing, open-cohort, international collaborative project between University of North Carolina and the Chinese Center for Disease Control and Prevention. The purpose of the CHNS is to investigate the impact of the Chinese government’s policies and programs related to family planning, health and nutrition. It also provides information about the health and nutritional status of the population during China’s economic transformation. The data mainly measure the impact of health behaviours and nutritional status and outcomes, as well as changes in demographic, family, economic and social factors.

The current project was conducted by an international research team; its members specialise in economics, public health, sociology, nutrition, Chinese studies and demography. We used a multistage random cluster to sample 7200 households and more than 30,000 residents in 15 provinces within 7 days. Our research is based on the data of 1891 children and their parents; it includes actual individual food consumption records, height, weight and regional distribution. We set the minimum age for the children in the study to one year, to eliminate skewed results caused by the nutritional impact of pregnancy [26]. Thus, we excluded families without children between the ages of one and eighteen. Children in the target age group were divided into three groups: those between one and six years old were classified as young children, who need be taken care of by their elders; those between seven and twelve years old were in the primary school period; and those between thirteen and eighteen years old were in the middle or high school period.

Individual food consumption was recorded by each meal for three consecutive days; we divided the food consumed [27] into ten main categories, according to the recommendations for food intake presented in the Dietary Guidelines for Chinese Residents (RFIDGCR), which was established by China’s Ministry of Health to provide residents with an intuitive way of understanding of what and how much food they should be eating (e.g., grains, meat, vegetables, fruit, dairy products, beans, eggs, fish, snacks and drinks). We counted the number of times the participants ate each type of food over the course of three days to determine the frequency of individual food consumption. We then combined the number of times participants ate each type of food with the weight of that food to determine the total average consumption of each type of food per day. Finally, we focused on individual food consumption, height, weight, gender and region, based on 2011 CHNS micro-data.

### Analysis methods

Statistical description and correlation analysis were used to determine the relationship between children’s overweight and obesity and parental food intake. We assumed that a household is composed of a mother (M), a father (F) and a child (C), based on the ‘one-child policy’ introduced by the Chinese government in 1980. Hursti demonstrated that parents play an important role in the formation of the dietary habits and preferences of their young children, and the earlier experiences of a particular food are the major determinants of the development of children’s dietary acceptance patterns [28]; as such, the relationships between parents and child were defined as *father–child* (F-C) and *mother–child* (M-C). The area of residence was divided into *rural* and *urban*, according to the Chinese household registration system. Household food consumption was recorded based on actual food consumption.

Currently, children’s nutritional status is mainly determined by the presence of the phenomena of overweight and obesity. These two variables are the most common measures of children’s nutritional status, referenced in the World Health Organization (WHO) Child Growth Standards indicator of body mass index-for-age and Z-scores (BMIZ) [29]. All the standardised BMIZ scores were determined by age and gender, using the world population health survey averages (i.e., the WHO 2006 child growth standards). In this manner, children with a BMIZ > 1 standard deviation (SD) were defined as *overweight*, and children with a BMIZ > 2 SD were defined as *obese*. From these definitions, we can see that the category *overweight* includes the category *obese*.

Data were analysed using Stata version 14 (StataCorp, College Station, TX, USA) to study the relationship between parental dietary patterns and children’s overweight and obesity. First, we conducted a descriptive analysis of the frequency of food consumption by parents and children, and we used children’s BMIZ scores to determine their level of overweight or obesity. These results are presented in Table 1 and Table 2. Second, we screened the data to assess the correlations between parents’ and children’s eating frequency, using a series of Pearson correlation analyses. Finally, we used a series of partial correlation analyses to find the correlations between parental food frequency and children’s BMIZ scores, researching how these relationships change with children’s age and with regional differences.

**Table 1:** The average weight of food consumed and proportion of each category of food consumed

Foods	Child			Father			Mother		
	Mean, g/day	Sd	Freq	Mean, g/day	Sd	Freq	Mean, g/day	Sd	Freq
Grain	271.96	167.20	1.00	454.67	239.85	1.00	356.50	190.80	1.00
Vegetable	224.26	166.80	0.99	345.32	170.54	1.00	323.40	171.60	1.00
Fruit	143.47	122.00	0.63	164.68	138.04	0.52	168.50	139.90	0.59
Meat	93.75	73.14	0.93	129.00	91.83	0.94	104.40	77.67	0.92
Been	54.47	50.82	0.65	80.97	71.69	0.67	73.64	66.77	0.66
Fish	50.12	39.88	0.44	73.92	57.72	0.48	62.05	46.80	0.47
Egg	42.77	30.87	0.73	43.67	31.57	0.69	41.84	29.40	0.69
Dairy	163.25	115.00	0.38	121.44	76.39	0.15	125.00	78.35	0.19
Drink	152.12	172.50	0.19	260.85	304.50	0.17	156.90	208.70	0.08
Snack	90.68	79.11	0.61	111.53	102.40	0.44	89.85	75.09	0.46

**Table 2:** Proportion of various shape of children

Body type category	Urban		Rural	
	Number	Percentage	Number	Percentage
Overweight	182	26.69	206	21.71
Obesity	92	13.48	102	10.75

## Results

### Sample characteristics of individuals’ food consumption

From the eligible age cohort, we obtained samples of 1631 children, 1334 fathers and 1555 mothers from the CHNS. The parents in the study range in age from 19 to 55 years old. Food consumption statistics (Table 1) show that more than 90% of participants—both children and parents—eat grains, meat and vegetables every day. Only a small portion of participants eat foods from all categories every day, and the proportion of parents who consume dairy daily is extremely low (15 and 19%, respectively). However, the proportion of children who consume drinks and dairy daily is higher (38%). In addition, nearly half of parents and children have a habit of snacking, which represents a significant change from previous dietary surveys [30]. Based on the weight of food consumed daily, paternal consumption of grains and meat and maternal consumption of meat exceed the recommended daily values of grains (250–400 g) and meat (50–75 g) (Exceeded food intake displayed in bold font) provided by the RFIDGCR. However, parental consumption of fruit and dairy fall below the daily recommended values of fruit (200–400 g) and dairy (300 g) (Insufficient food intake displayed in italic font) provided by RFIDGCR. In general, parental food intake is relatively balanced, meeting the requirements of the RFIDGCR.

### Sample characteristics of children’s overnutrition

**Fig. 1:**
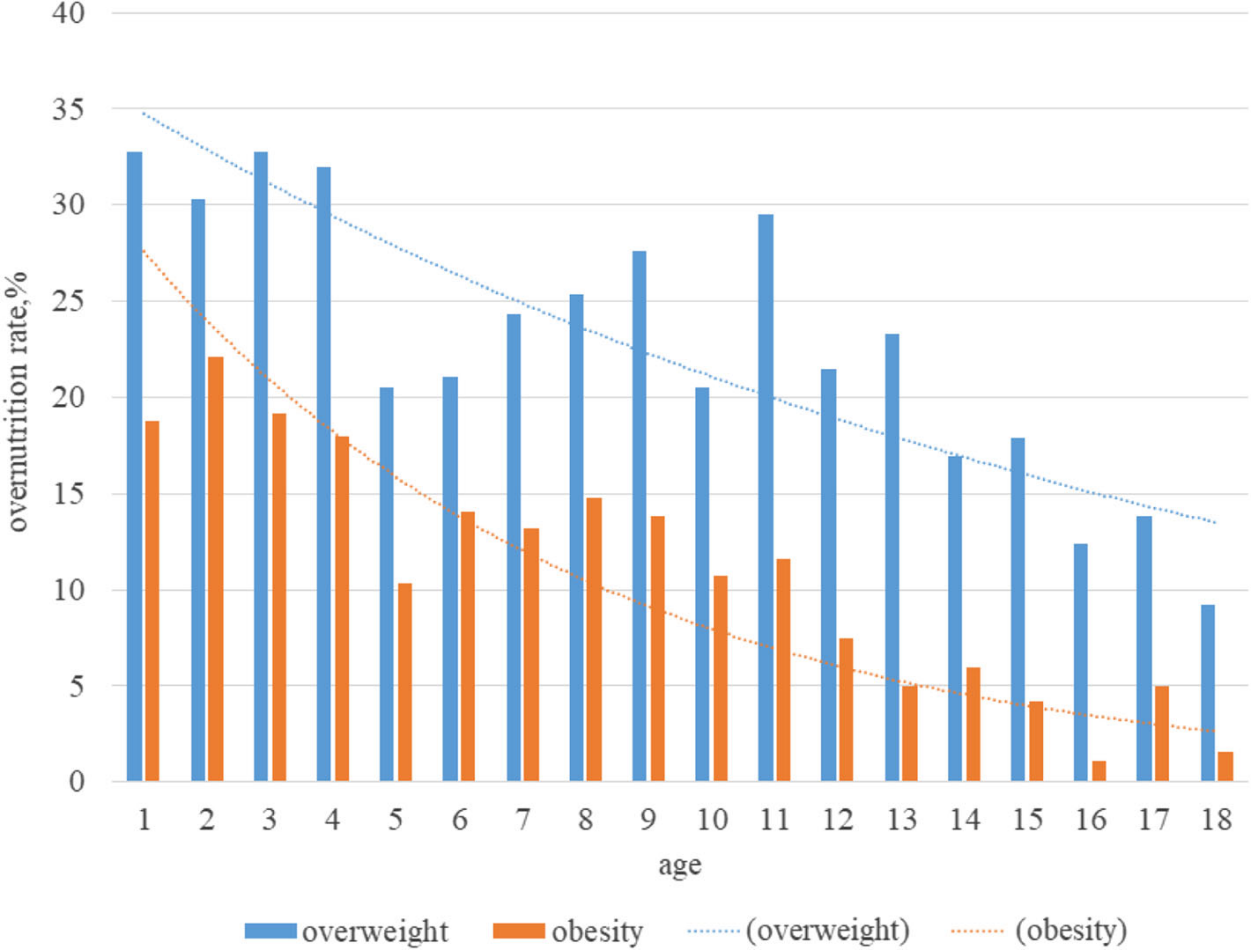
Average overnutrition rates among children of different ages

After determining individual food consumption, we measured children’s overnutrition rates in rural and urban areas in the categories of *overweight* and *obese* (Table 2). It is worrying that both overweight and obesity are high among children in both urban and rural regions. The proportions of overweight and obese urban children are 26.69 and 13.48%, respectively. In contrast, the proportions of overweight and obese rural children are 21.71 and 10.75%, respectively—on average, 4% lower than the same proportions among urban children. Based on the difference in average overnutrition rates according to children’s age (Fig. 1), we found that rates of both overweight and obesity are higher in young children and lower in those in more advanced adolescence. More specifically, at age one, 32.8% of children are overweight, 21.5% are overweight at age twelve and 9.23% are overweight by adulthood. Children’s obesity rates also decrease with age, falling from 18.8% at one year old to 1.54% at eighteen years old. The rates of all types of overnutrition tend to decrease with age, but the proportion of overnutrition remains very high compared with the nutritional status of children during the early period of Chinese economic reform [31].

### Correlation between parent’s and children’s eating behaviours

Table 3 demonstrates the results of the correlation analysis between parents’ and children’s food consumption. In this analysis, the parent–child correlation for each type of food was calculated using the methods described above. All the interrelationships between parent’s and children’s eating behaviours were found to be positive and highly statistically significant (*p* < 0.01), and the correlations of eating behaviours between mothers and their children were higher than those between fathers and their children (except for the category of drinks). When considering the types of food, most of the food intake categories consumed by mothers were positively correlated with those of children (estimated coefficient > 0.5), but such relationships existed only in some categories of food consumed by fathers. This provides strong evidence of the need for more studies on the effects of parental dietary behaviours on children’s nutritional status.

**Table 3:** Correlations between parental and children’s eating behaviours

Parental food correlation	M-C	F-C
Grain	0.498***	0.464***
Vegetable	0.430***	0.359***
Fruits	0.642***	0.592***
Meat	0.567***	0.506***
Been	0.508***	0.484***
Fish	0.665***	0.573***
Egg	0.606***	0.523***
Dairy	0.518**	0.381***
Drink	0.373***	0.431***
Snack	0.519***	0.444***

*** *p* < 0.01

### Effect of age on childhood overweight and obesity

Table 4 and Table 5 show the correlation between parental eating behaviours and the overweight and obesity rates of children of different ages. Table 6 further shows the correlation merged mother and father together, according to the length of time they spend preparing food, aiding in the determination of the total average parental effect. For example, if a mother consumes 300 g of food and takes 60 min to prepare the food, and a father consumes 400 g of food and takes 45 min to prepare the food, the total average food intake of parents is 342.86 g ([300*60 + 400*45]/[60 + 45] = 342.86). The consumption of fish, beans, dairy and drinks were not considered because these types of food do not have a significant correlation with childhood overweight and obesity.

**Table 4:** Correlations between paternal eating behaviours and children’s overweight and obesity at different ages

Age	Status	Grain	Vegetable	Fruit	Meat	Egg	Snack
1–18	Overweight	−0.063*	− 0.055*	− 0.053	− 0.010	0.041	0.048
	Obesity	−0.058*	−0.009	− 0.011	0.030	0.072*	0.031
1–6	Overweight	−0.93*	−0.037	0.020	0.013	−0.019	0.92*
	Obesity	−0.074*	0.012	0.022	0.054	−0.032	0.041
7–12	Overweight	−0.075*	−0.891*	− 0.107*	−0.063*	0.127*	0.021
	Obesity	−0.045*	0.012	0.03	0.052	0.159*	0.019
13–18	Overweight	−0.044	− 0.016	−0.002	0.031	0.032	0.066
	Obesity	−0.027	−0.032	− 0.132*	0.015	0.065	0.039

* *p* < 0.1

**Table 5:** Correlations between maternal eating behaviours and children’s overweight and obesity at different ages

Age	Status	Grain	Vegetable	Fruit	Meat	Egg	Snack
1–18	Overweight	−0.079*	− 0.042*	0.022	− 0.043*	0.032	0.050
	Obesity	−0.043*	0.025	0.033	0.009	0.090*	0.041
1–6	Overweight	−0.125*	−0.069*	0.018	−0.013	0.081	0.815*
	Obesity	−0.086*	−0.018	0.06	0.008	0.066	0.062
7–12	Overweight	−0.079*	−0.082*	− 0.058	−0.047*	0.185*	0.103
	Obesity	−0.006	0.046	0.047	0.027	0.158*	0.093
13–18	Overweight	0.058	−0.031	−0.007	− 0.002	0.074	0.037
	Obesity	−0.046*	−0.013	− 0.015	−0.011	0.043	0.008

* *p* < 0.1

**Table 6:** Correlations between parental eating behaviours and children’s overweight and obesity at different ages

Age	Status	Grain	Vegetable	Fruit	Meat	Egg	Snack
1–18	Overweight	−0.105*	− 0.028*	0.008	− 0.042*	0.116	0.074
	Obesity	−0.085*	− 0.011	0.01	−0.026	0.091*	0.066
1–6	Overweight	−0.143*	0.001*	0.004	−0.004	0.103	0.093*
	Obesity	−0.137*	0.016	0.025	0	0.096	0.090
7–12	Overweight	−0.103*	−0.035*	− 0.031*	−0.071*	0.163*	0.098
	Obesity	−0.047	−0.016	0.028	−0.03	0.136*	0.097
13–18	Overweight	−0.058	−0.061	0.001	−0.028	0.086	0.018
	Obesity	−0.061*	−0.058	− 0.017	−0.033	0.049	−0.019

* *p* < 0.1

The paternal consumption of grains and vegetables was found to have a significant negative correlation with children’s overweight, while the paternal consumption of grains and eggs was found to have a positive significant correlation with obesity. There is no significant relationship between children’s overweight and obesity and the paternal consumption of other types of food. In children between the ages of one and six, the paternal consumption of grains has a significant negative correlation with children’s overweight and obesity. However, these correlations begin to change as children grow up. For example, the category of snacks yields a particularly significant positive correlation with children’s obesity as children increase in age. In addition, for children between the ages of seven and twelve, the correlation between the paternal consumption of fruit, meat and eggs and children’s overweight undergoes a significant positive in comparison to those younger than seven; in contrast, the correlation between the paternal consumption of grains and vegetables has the same influence on the overnutrition of children aged seven to twelve when compared to younger children, with just a slight change in the size of the coefficient. In children ages 13 to 18, the correlation between the paternal eating behaviours and children’s overweight and obesity begins to weaken, and the paternal consumption of only a few types of food were associated with children’s overweight and obesity. In conclusion, the correlation between paternal eating behaviours and children’s overweight and obesity is much more significant during children’s younger years, beginning to weaken as children grow older. These significant correlations prove that the paternal consumption of several types of foods has obvious effects on children’s overnutrition [3].

The maternal consumption of grains, vegetables and meat has a significant negative correlation with children’s overweight, but the maternal consumption of grains and eggs has a positive significant correlation with obesity. No significant relationship was found between children’s overweight and obesity and the maternal consumption of other types of food. This is a similar association to that found for paternal dietary patterns. For children between the age of one and six, the maternal consumption of grains and vegetables was found to have the same influence on children’s overweight and obesity compared to that of fathers, but the correlation coefficient is significantly higher. The maternal consumption of snacks was found to have a significant positive correlation with children’s obesity. In children between the ages of seven and twelve, the correlation between the maternal consumption of grains, vegetables and meat and children’s overweight had a negative significance, and the maternal consumption of eggs had a positive significant correlation with children’s overweight and obesity. For children between the ages of 13 and 18, the correlation between maternal eating behaviours and children’s overweight and obesity also weakens in comparison to that of younger children, with only the maternal consumption of grains affecting children’s obesity. The correlation between maternal eating behaviours and children’s overweight and obesity is therefore most obvious children’s younger years, and it begins to weaken as children grow up. These significant correlations prove that the maternal consumption of several types of food has obvious effects on children’s overnutrition.

Considering the average food consumption of parents in relation to the length of cooking time, the changing trends of the correlations between parental eating behaviours (i.e., those of both the mother and father) and children’s overweight and obesity are approximately similar to those of the father and mother separately, shown in Table 6. The correlation between parental eating behaviours and children’s overweight and obesity is most obvious when children are younger, and it begins to weaken as children get older.

These maternal and paternal eating behaviours have approximately the same significant negative or positive correlations with children’s overweight and obesity, with just small differences in the size of the correlation coefficient for each type of food. The influence of both paternal and maternal dietary behaviours on children’s overweight and obesity in young children is most significant, and the influence weakens during adolescence. While the relationship and coefficient between the parental consumption of some types of food and children’s overweight and obesity becomes less significant as children’s age increases, that of other types of food becomes more significant as children’s age increases. In any case, these correlations between parental intake of various types of food and children’s overweight and obesity are significant, meaning that it is vital that parents pay attention to their own eating behaviours to help their children develop healthy eating behaviours and avoid overnutrition, especially in young children.

### Effects of region on children’s overweight and obesity

Table 7 shows the correlation between parental eating behaviours and the overweight and obesity of children in urban and rural areas. The consumption of fish, beans, dairy, fruit and snacks is not considered, s these types of food do not have a significant correlation with children’s overweight and obesity.

**Table 7:** Correlations between parental eating behaviours and children’s overweight and obesity in different regions

Object	Region	Status	Grain	Vegetable	Meat	Egg	Drink
F-C	Urban	Overweight	− 0.024	− 0.013	0.012	0.068	0.322*
		Obesity	−0.065*	0.012	0.062*	0.062	0.241*
	Rural	Overweight	−0.083*	− 0.082*	− 0.079*	− 0.006	−0.112
		Obesity	−0.068*	−0.025	− 0.033	0.031	− 0.080
M-C	Urban	Overweight	−0.014	−0.014	− 0.011	0.074*	0.058
		Obesity	−0.027	0.037	0.013	0.084*	0.064
	Rural	Overweight	−0.132*	− 0.084*	−0.093*	0.034	−0.062
		Obesity	−0.093*	−0.032	− 0.068*	0.034	− 0.121

* *p* < 0.1

The differences in the relationship between parental eating behaviours and children’s overweight and obesity according to region are considerably significant. The correlation between the parental consumption of staple foods and children’s overweight and obesity in rural areas was found to be significantly greater than was the correlation between the parental consumption of staple foods and children’s overweight and obesity in urban areas, regardless of the size of the correlation coefficient or the types of food consumed. In urban regions, the paternal consumption of grains was found to be significantly negatively associated with children’s obesity, but the paternal consumption of meat was found to have the opposite significant association.

Additionally, only the paternal consumption of drinks is positively correlated with children’s overweight and obesity. In contrast, only the maternal consumption of eggs is significantly positively associated with children’s overweight and obesity. The maternal consumption of grains, vegetables and meat has a negative influence on children’s overweight and obesity in rural areas but not in urban areas. In general, parental eating behaviours and children’s overweight and obesity are more closely associated in rural areas than they are in urban areas, but the consumption of eggs and drinks only has significant correlations in urban areas. These significant relationships vary by region, and the effects of parental and maternal eating behaviour on children’s overweight and obesity are not homogeneous.

## Discussion

The results of this study demonstrate that parental eating patterns are significantly associated with children’s nutritional status; however, these associations change based on children’s age and on region. Moreover, some types of food have a positive effect, while other types of food have a negative effect. As such, parents must model different dietary patterns to different children.

In terms of children’s nutritional status by age, the most significant correlation change was found to be that of the parental consumption of snacks and young children’s overweight and obesity. The correlation coefficient and influence direction between the parental consumption of other types of food and children’s overweight and obesity, however, change with children’s age. These changes can be explained by China’s unique culture of childcare. Young children are often taken care of and fed by their parents, which is why the correlation between parental eating behaviours and young children’s overnutrition are more significant than are those of older children [32]. In addition, there is an old Chinese saying, ‘if you can eat, you are blessed’ [33]; some parents worry about ensuring that their child’s nutritional status matches that of their peers, believing that it is a blessing to eat more. As such, they give their children a lot of food, resulting in higher rates of obesity and overweight in young children. The influence of parental eating behaviours on adolescents is weaker than that on younger children. This weakening trend is slightly evident among children ages seven to twelve but becomes more evident among children between the ages of thirteen and eighteen. This could be because children in the younger age group are required to eat lunch in their primary school because of the School Lunch Program implemented in China [34]. Therefore, as the amount of time spent dining with parents is reduced, the association of parents’ eating behaviours begins to weaken. Then, many children between ages thirteen and eighteen attend boarding school, returning home only on weekends; this means that they have dinner with their parents even less frequently, thus weakening the correlations between parental eating behaviour and children’s overweight and obesity [35]. The fact that the effect of parental eating behaviours on children’s overweight and obesity changes as children’s age increases is a novel finding. In addition, the rate of obesity and overweight in children decreases as they age. This suggests that parents may be less conscious of nutrition than they are of adhering to the traditional Chinese cultural phenomenon of doting on children, thus having a poor influence on their children’s nutritional habits. The percentage of fathers and mothers who have knowledge of the RFIDGCR when their children are infants has been shown to be 29.67 and 30.56%, respectively, and this increases significantly by the time children are 13 to 18 years old (to 33.58 and 32.39%, respectively). Therefore, parental dietary knowledge needs to be further improved to ensure that they can help their children maintain a healthy body from birth [36].

The different correlations between parental consumption of staple foods and children’s overnutrition are more obvious when comparing rural and urban areas. The maternal and paternal consumption of grains, vegetables and meat is significantly correlated with children’s overweight and obesity in rural areas, but only the paternal consumption of grains and meat is significantly correlated with children’s obesity in urban areas. In addition, the parental consumption of eggs and drinks only has a positive influence on children’s overweight and obesity in urban areas. The influence of parents’ dietary behaviours on children is, therefore, significantly stronger in rural areas than it is in urban areas. Also, urban areas showed a higher correlation with calorie-dense foods. This may be attributed to regional distributions and economic structures [37]. In urban areas of China, residents have higher living standards; they can buy any food they want to buy, and their consumption of drinks and snacks is also increasing rapidly because of the impact of Western dietary patterns and globalisation [38, 39]. Furthermore, some urban schools also offer a variety of foods to children who eat at school [40]. As a result, the diet patterns of those living in urban areas are more diverse than are those of people living in rural areas. As a result, childhood overweight and obesity in urban populations is not as closely associated with the dietary patterns of parents. In contrast, residents of rural China have lower living standards and limited access to amenities. Their main sources of food—grains, vegetables and meat—come from farming, and many are self-sufficient [41], thus leading to a monotonous diet. In addition, a high proportion of rural students must bring a week’s worth of food from home with them to school, leading to a repetitive diet of primarily rice, a few vegetables and pork [42]. Data on children’s food consumption show that the mean grain consumption of children in rural areas is 272.75 g/day, which is much higher than that of children in urban areas (242.11 g/day). Diversified food patterns can spread the risk—not unlike a wealth management product that is composed of many stocks and bonds—thus weakening the relationship between parents’ food patterns and children’s nutritional status. Therefore, parental food patterns have a significant correlation with children’s overweight and obesity because parental dietary patterns tend to be less diverse in rural areas. Arimond and Ruel’s [43, 44] research revealed the positive association of dietary diversity on children’s health status. The Chinese government must improve the quality of children’s diets in rural areas by diversifying their food intake. Of course, socioeconomic status and demographic factors also play an important role in children’s overweight and obesity, especially in relation to drinks, snacks and sugar. This may contribute to the higher levels of overweight and obesity found among children in urban areas when compared to those in rural areas. In addition, children in rural areas generally perform more physical activity as a result of doing farm work, having less time to watch television and consuming less high-calorie food when compared to those in urban areas; this could also explain the differences in children’s overweight and obesity [45]. It is worth noting that although overweight rates in rural areas are lower than those in urban areas, this does not mean that low food availability and low living standards are good for children; such conditions may simply cause other serious malnutrition phenomena, such as stunting, wasting, underweight and insufficient protein intake [46].

The current research, based on cognitive theory, provides evidence of the associations between parental food patterns and children’s overnutrition to obtain a series of novel conclusions. The association between parental food patterns and the overnutrition of children decreases as children get older, with the strongest associations existing in young children and a negligible association being found in children between the ages of 13 and 18. In addition, parental food patterns have a much stronger association with children’s overnutrition in rural areas than they do in urban areas. There is no doubt that parental dietary patterns related to certain types of food do contribute to overweight and obesity in children, while those related to healthier foods positively contribute to children’s nutritional status. This research used actual individual food consumption data from China by focusing on two types of overnutrition (i.e., overweight and obesity) and ten categories of food. These conclusions provide useful, practical ways in which to promote the health of Chinese children. Of primary importance is the finding that we can predict—and therefore improve—children’s nutritional status by improving the eating behaviours of their parents, especially for young children and those in rural areas.

However, several limitations of this study do exist. This study does not demonstrate the association between the parental consumption of each type of food and children’s overweight or obesity; it only shows the correlation coefficients and significance between parents and children, focusing on the relationship between parental dietary patterns and children’s overweight and obesity. Many variables that did not meet the significance standard were not explained in detail in this paper, but it is helpful to use them as comparison variables to highlight the significant relationship between parental eating behaviours and children’s overnutrition, considering the differences based on children’s age and region. In addition, we did not conduct a relevant correlation analysis to test and discuss the possible effects of other family members on the nutritional status of the subjects studied in this paper; this decision was made because of the ‘one-child policy’. Children’s dietary behaviours are mainly influenced by their parents [28]; while other family members and surrounding environments may have a certain degree of influence on children, the present manuscript only considered the influence of parents’ dietary patterns. Furthermore, the present manuscript only conducted a series of correlation analyses between parental food patterns and children’s overnutrition. For the findings of the present research—such as the prevalence of obesity varying with age and region and the low consumption of dairy products and high consumption of meat products in China—causal analysis is necessary to further investigate the causes of children’s obesity in relation to the unique dietary culture of China.

The associations between parental eating behaviours and children’s overweight and obesity determined in this study provide a number of new insights about the effect of parental food patterns on children’s overnutrition under different conditions. Although some similar correlations have previously been found for children of different ages and in different regions, the current results extend previous parental role modelling results using evidence from actual individual food consumption and provide a comprehensive analysis of differences in children’s age and region. These new insights reveal how the associations between parental eating behaviours and children’s overweight and obesity change according to age and region and when considering the culture and population distribution of China. The association between parental food patterns and children’s overweight and obesity decreases as children get older. Therefore, because childhood obesity rates are particularly high in young children, parents must pay more attention to their own healthy eating behaviours, such as reducing the consumption of high-calorie snacks and drinks. For adolescents, parents must also pay attention to their meals and consume more fruits and vegetables themselves to reduce children’s overweight and obesity rates and improve children’s nutritional status. In rural areas, parental dietary patterns may be limited because diverse food needs are hard to meet, thus causing the effect of parental eating behaviours on children’s overweight and obesity to be more significant. Therefore, the Chinese government must focus on improving the diets of rural children while working to develop the rural economy [47]. For example, to address malnutrition caused by a monotonous diet and the lower living standards of rural areas, China has implemented a program called the Nutrition Improvement Program for Rural Compulsory Education Students, which is providing a nutritional allowance of three yuan a day for every rural compulsory education student. This program has helped to reduce the prevalence of childhood malnutrition. Based on the results of this study, it is suggested that more programs of this type throughout China would be effective.

## Conclusions

The present study demonstrated that parental dietary patterns have a significant correlation with children’s nutrition status, but the correlation varied considerably based on parental food categories, children’s age and region. The association between parental dietary patterns and children’s overweight and obesity is most strongly significant in young children, but it begins to weaken as children grown up. In addition, the association between parental dietary patterns and children’s overnutrition in rural areas is more significant than it is in urban areas. For young children and those in rural areas, care and policy providers should be aware of the differences in need of different groups of children. Parents should also be encouraged to improve their dietary knowledge, and healthy foods should be made more easily available, allowing parents to guide their children to develop good dietary behaviour and achieve a good nutritional status.

## Data Availability

The datasets used and/or analyzed during the current study are available from the corresponding author on request.

## Abbreviations

BMIZ: Body mass index- for- age Z-score
C: Child
CHNS: Data of China health and nutrition survey
F: Father
F-C: Father and child
M: Mother
M-C: Mother and child
RFIDGCR: Recommended food intake presented in the Dietary Guidelines for Chinese Residents
SD: Standard deviation
WHO: World health organization
